# Assessment of diagnostic accuracy of biomarkers to assess lung consolidation in calves with induced bacterial pneumonia using receiver operating characteristic curves

**DOI:** 10.1093/jas/skab368

**Published:** 2021-12-17

**Authors:** Miriam Martin, Michael D Kleinhenz, Shawnee R Montgomery, Dale A Blasi, Kelli M Almes, Angela K Baysinger, Johann F Coetzee

**Affiliations:** 1 Department of Anatomy and Physiology, Kansas State University College of Veterinary Medicine, Manhattan, KS, USA; 2 Department of Clinical Sciences, Kansas State University College of Veterinary Medicine, Manhattan, KS, USA; 3 Department of Animal Science, Kansas State University, Manhattan, KS, USA; 4 Department of Diagnostic Medicine/Pathobiology and Kansas State Veterinary Diagnostic Laboratory, Kansas State University College of Veterinary Medicine, Manhattan, KS, USA; 5 Merck Animal Health, De Soto, KS, USA

**Keywords:** biomarkers, bovine respiratory disease, lung lesion, pain

## Abstract

Bovine respiratory disease (**BRD**) is the most economically significant disease for cattle producers in the U.S. Cattle with advanced lung lesions at harvest have reduced average daily gain, yield grades, and carcass quality outcomes. The identification of biomarkers and clinical signs that accurately predict lung lesions could benefit livestock producers in determining a BRD prognosis. Receiver operating characteristic (**ROC**) curves are graphical plots that illustrate the diagnostic ability of a biomarker or clinical sign. Previously we used the area under the ROC curve (**AUC**) to identify cortisol, hair cortisol, and infrared thermography imaging as having acceptable (AUC > 0.7) diagnostic accuracy for detecting pain in cattle. Herein, we used ROC curves to assess the sensitivity and specificity of biomarkers and clinical signs associated with lung lesions after experimentally induced BRD. We hypothesized pain biomarkers and clinical signs assessed at specific time points after induction of BRD could be used to predict lung consolidation at necropsy. Lung consolidation of > 10% was retrospectively assigned at necropsy as a true positive indicator of BRD. Calves with a score of < 10% were considered negative for BRD. The biomarkers and clinical signs analyzed were serum cortisol; infrared thermography (IRT); mechanical nociceptive threshold (**MNT**); substance P; kinematic gait analysis; a visual analog scale (**VAS**); clinical illness score (**CIS**); computerized lung score (**CLS**); average activity levels; prostaglandin E_2_ metabolite (**PGEM**); serum amyloid A; and rectal temperature. A total of 5,122 biomarkers and clinical signs were collected from 26 calves, of which 18 were inoculated with *M. haemolytica*. All statistics were performed using JMP Pro 14.0. Results comparing calves with significant lung lesions to those without yielded the best diagnostic accuracy (AUC > 0.75) for right front stride length at 0 h; gait velocity at 32 h; VAS, CIS, average activity and rumination levels, step count, and rectal temperature, all at 48 h; PGEM at 72 h; gait distance at 120 h; cortisol at 168 h; and IRT, right front force and serum amyloid A, all at 192 h. These results show ROC analysis can be a useful indicator of the predictive value of pain biomarkers and clinical signs in cattle with induced bacterial pneumonia. AUC values for VAS score, average activity levels, step count, and rectal temperature seemed to yield good diagnostic accuracy (AUC > 0.75) at multiple time points, while MNT values, substance P concentrations, and CLS did not (all AUC values < 0.75).

## Lay Summary

Bovine respiratory disease (BRD) is the most economically significant disease for cattle producers in the United States, affecting 16.2% of cattle on feed. Cattle with advanced lung lesions at harvest have reduced average daily gain, yield grades, and carcass quality outcomes. The identification of biomarkers and clinical signs that accurately predict lung lesions could benefit livestock producers in determining a BRD prognosis. Herein, we used receiver operating characteristic curves to assess the predictive value of biomarkers and clinical signs associated with lung lesions after experimentally induced BRD. In the first 72 h after onset of BRD, right front stride length, gait velocity, visual analog scale score, clinical illness score, average activity level, step count, and rectal temperature yielded the best diagnostic accuracy (AUC > 0.75) for predicting calves with significant lung lesions (>10% consolidation) at necropsy. Biomarkers and clinical signs with the best diagnostic accuracy early in the disease process would likely be the most valuable in field conditions. These results can be used to guide refinement of the optimal time points and biomarkers for the diagnosis of significant lung lesions after BRD.

## Teaser

Receiver operating curve analysis can be a useful indicator of the predictive value of pain biomarkers in cattle with induced bacterial pneumonia. Area under the curve values for visual analog scale score, average activity levels, step count, and rectal temperature seemed to yield good diagnostic accuracy at multiple time points in the disease process.

## Introduction

Bovine respiratory disease (**BRD**) is the most economically significant disease for cattle producers in the United States, affecting 16.2% of cattle on feed. Fibrinous pleuropneumonia produced by *M. haemolytica* and is the most common form of acute pneumonia in weaned, stressed calves (Andrews and Kennedy, 1997). Pleuritis is an indication of the aggressiveness of the lung infection and the extent of infection and inflammation; lipopolysaccharide and leukotoxin are the two factors responsible for the destructive lesions of *M. haemolytica* infection. (Panciera and Confer, 2010). Lung lesion incidence has been recorded at 62% to 67% (Schneider et al., 2009; Blakebrough-Hall et al., 2020; Tennant et al., 2014). A biomarker is a molecular, histologic, or physiological characteristic “that is an indicator of normal biological processes, pathogenic processes, or responses to an exposure or intervention, including therapeutic interventions” (FDA, 2017). The relationship between clinical illness score, computerized lung score, rectal temperature, facial thermography, behavior and lung lesion scores has been examined in past studies to detect the presence of BRD (White et al., 2008; DeDonder et al., 2010; Baruch et al., 2019). However, which biomarkers and clinical signs yield good diagnostic accuracy when predicting lung lesions at varying time points throughout the disease state has not been examined.

Receiver operating characteristic (**ROC**) curves are graphical plots that illustrate the diagnostic ability of a test as its discrimination threshold is varied. The plot of true positive (sensitivity) verses false positive (1-specificity) across possible cutoff values generates a ROC curve (Hajian-Tilaki, 2013). The area under the ROC curve (**AUC**) can be used to measure discriminative ability. The objective of this analysis was to use AUC values derived from ROC analysis to assess the predictive value of BRD biomarkers and clinical signs to evaluate the presence of lung lesions after experimentally induced bacterial pneumonia.

## Materials and Methods

This study was reviewed and approved by the Institutional Animal Care and Use Committee at Kansas State University (IACUC# 4465).

### Study design

Twenty-six calves, 6 to 7 months of age, weighing an average of 185 ± 4 kg were enrolled onto the study. On arrival to the study site, calves were affixed with a three-axis accelerometer ear-tag (Allflex Livestock Intelligence, Madison, WI) to quantify activity. At 24 h prior to the start of the study, 18 calves were inoculated with a strain of *Mannheimia haemolytica* using bronchoalveolar lavage as described by Theurer et al. (2013). The right apical lung lobes were inoculated using broncho-selective endoscopy. A 10-mL dose of *M. haemolytica* serotype A1 at 1 × 10^9^ cfu/mL was used to inoculate each calf, then the endoscope was flushed with 60 mL of phosphate-buffered solution to achieve a total volume of 70 mL. The eight control calves received 70 mL of phosphate-buffered solution as described above and were all inoculated prior to the calves inoculated with *M. haemolytica*. At any point while on study, if the rectal temperature of a calf was >40.3 °C, tildipirosin was administered at a dosage of 4 mg/kg. Eight calves that were inoculated with *M. haemolytica* were administered flunixin transdermal at a dosage of 3.33 mg/kg at 0 and 24 h relative to disease onset. Across all treatments, calves were restrained, and samples were collected the same number of times.

Disease onset was considered to be 24 h following inoculation and was considered time point 0 h. Biomarker and clinical sign variables were collected at −48 prior to disease onset, 0 (disease onset), 4, 8, 24, 32, 48, 72, 96, 120, 144, 168, and 192 h relative to disease onset, in addition to the three-axis accelerometer ear-tags continuously collecting data throughout the study. A total of 5,122 biomarkers and clinical signs were collected which included: infrared thermography (**IRT**) imaging, kinematic gait analysis, mechanical nociception threshold (**MNT**), visual analog scale (**VAS**) score, clinical illness score (**CIS**), computerized stethoscope (Whisper Veterinary Stethoscope, Merck Animal Health, Madison, NJ) lung score (**CLS**), average activity levels, rectal temperature, and blood sampling for cortisol, substance P, prostaglandin E_2_ metabolite (**PGEM**), and serum amyloid A (**SAA**) analysis. All trained evaluators were blinded to treatment for the duration of the study. Following the 192 h collection, calves were euthanized and transported to the Kansas State Veterinary Diagnostic Laboratory for necropsy and lung lesion scoring. The postmortem examination was performed by a board-certified veterinary pathologist (K.M.A.) to determine lung lesions and a lung lesion score was assigned based on lung consolidation. The lung lesion score was determined using methods described by Fajt et al. (2003). Briefly, the extent of consolidated lung divided by the total lung volume was determined. A lung score > 10% was used as a positive indicator of BRD for the sake of this analysis. Regardless of therapeutic intervention, calves were grouped based on a lung score > 10% or < 10%. The biomarkers and sample collection time points are outlined in Table 1.

**Table 1.** Collection time points for each BRD biomarker with 0 h being time of disease onset

Biomarker^1^	Time point
Cortisol, h	0, 4, 8, 24, 32, 48, 72, 96, 120, 144, 168, 192
IRT, h	0, 4, 8, 24, 32, 48, 72, 96, 120, 144, 168, 192
MNT, h	8, 24, 72, 192
Substance P, h	0, 4, 8, 24, 32, 48, 72, 96, 120, 144, 168, 192
Kinematic gait analysis, h	0, 4, 8, 24, 32, 48, 72, 96, 120, 144, 168, 192
VAS, h	0, 4, 8, 24, 32, 48, 72, 96, 120, 144, 168, 192
CIS, h	0, 4, 8, 24, 32, 48, 72, 96, 120, 144, 168, 192
Computerized lung score, h	0, 4, 8, 24, 32, 48, 72, 96, 120, 144, 168, 192
Average activity, h	0, 24, 48, 72, 96, 120, 144, 168
Average rumination, h	0, 24, 48, 72, 96, 120, 144, 168
Step count, h	0, 24, 48, 72, 96, 120, 144, 168
PGEM, h	0, 72, 192
Serum amyloid A, h	0, 4, 8, 24, 32, 48, 72, 96, 120, 144, 168, 192
Rectal temperature, h	0, 8, 24, 32, 48, 72, 96, 120, 144, 168

IRT, infrared thermography; MNT, mechanical nociceptive threshold; VAS, visual analog scale; CIS, clinical illness score; PGEM, prostaglandin E_2_ metabolites.

### Physiological and behavioral parameters

#### Serum cortisol

A total of 312 cortisol samples made up the data set. The samples were obtained as described by Kleinhenz et al. (2017). Blood was obtained by jugular venipuncture using a syringe (Kleinhenz et al., 2018; 2019b) and was immediately transferred to a blood tube and centrifuged at 3,000 g for 10 min. The serum was pipetted into cryovials, placed on dry ice, and stored at −80 °C until analysis. Cortisol concentrations were determined using a commercially available radioimmunoassay (MP Biomedicals, Santa Ana, CA).

#### Infrared thermography

A total of 312 IRT images made up the data set. Mean and percent change from baseline values from images of the medial canthus of the left eye were included in the analysis. Infrared thermography images were obtained using a research-grade infrared camera (Fluke TiX580, Fluke Corp, Everett, WA). The IRT camera was calibrated prior to being used with the ambient temperature and relative humidity. As described in Kleinhenz et al. (2017) and Kleinhenz et al. (2018), an image of the lateral aspect of the head was obtained so that the image contained the medial canthus of the eye. Infrared images were analyzed using research-grade computer software (SmartView v. 4.3, Fluke Thermography, Plymouth, MN).

#### Mechanical nociception threshold

A total of 208 MNT measures made up the data set. Using a hand held pressure algometer (Wagner Instruments, Greenwich, CT), force was applied perpendicularly at a rate of approximately 1 kg of force per second at one location on each side of the ribs of each calf over the sixth intercostal space for a total of two locations, as described in (Williams et al., 2020). A withdrawal response was indicated by an overt movement away from the applied pressure algometer and values were recorded by a second investigator to prevent bias. Locations were tested three times in sequential order, and the values were averaged for statistical analysis.

#### Substance P

A total of 312 substance P samples made up the data set. As described in Kleinhenz et al. (2017), benzamidine hydrochloride (final concentration of 1mM) was added to ethylenediaminetetraacetic acid (**EDTA**) blood tubes (BD Vacutainer, Franklin Lakes, NJ) 48 h prior to the start of the studies. During sample collection, 3 mL of blood was added to the spiked EDTA tube. The samples were immediately placed on ice, centrifuged within 30 min of collection, and the plasma was placed into cryovials. The cryovials were stored at −80 °C until analysis. Substance P levels were determined using the methods described by Van Engen et al. (2014) using nonextracted plasma.

#### Kinematic gait analysis

A total of 2,184 gait readings made up the data set. As described in Kleinhenz et al. (2019c), a commercially available kinematic gait system (Walkway, Tekscan, Inc., South Boston, MA) was used to record gait and biomechanical parameters. The gait system was calibrated, using a known mass, daily and before each use of the computer software to ensure accuracy of the measurements at each time point. Video synchronization was used to ensure consistent gait between and within calf at each time point. Using research specific software (Walkway 7.7, Tekscan, Inc.), force, contact pressure, and impulse were assessed.

#### Visual analog scale

A total of 312 VAS scores made up the data set. As described in Martin et al. (2020a), a daily VAS pain assessment was conducted by two trained evaluators blinded to treatment allocations. The VAS used was a 100-mm (10 cm) line anchored by descriptors of “No Pain” on the left (0 cm) and “Severe Pain” on the right (10 cm). Five parameters were used to assess pain: depression, tail swishing or flicking, stance, head carriage, and foot stomping or kicking. No pain was characterized by being alert and quick to show interest, no tail swishing, a normal stance, head held above spine level, and absence of foot stomping. Severe pain was characterized by being dull and showing no interest, more than three tail swishes per minute, legs abducted, head held below spine level, and numerous stomps. The evaluator marked the line between the two descriptors to indicate the pain intensity. A millimeter scale was used to measure the score from the zero anchor point to the evaluator’s mark. The mean VAS measures of the two evaluators were combined into one score for statistical analysis.

#### Clinical illness score

A total of 312 CIS measures made up the data set. A CIS was assigned by two trained evaluators blinded to treatment allocations. The CIS consisted of (1) is a normal healthy animal, (2) slightly ill with mild depression or gauntness, (3) moderately ill demonstrating severe depression/labored breathing/nasal or ocular discharge, and (4) severely ill and near death showing minimal response to human approach. If either evaluator scored a calf > 1, that score was used for statistical analysis, with 1 being considered normal and greater than 1 considered abnormal.

#### Computerized lung score

A total of 312 computerized lung scores made up the data set. A computerized stethoscope (Whisper, Merck Animal Health, De Soto, KS) was used to analyze lung and heart sounds via a machine-learning algorithm that assigns a lung score from 1 to 5, with 1 being normal and 5 being severely compromised lung tissue (Nickell et al., 2020). The bell of the lung stethoscope was placed approximately two inches caudal and dorsal to the right elbow of each calf, and lung sounds were recorded for 8 s. If the recording was deemed acceptable by the computer program, the score was recorded.

#### Average activity and rumination

A total of 208 average activity and rumination scores made up the data set. A three-axis accelerometer ear-tag (Allflex Livestock Intelligence, Madison, WI) was used to quantify activity and rumination throughout the study. The average number of active and ruminating minutes over 60 min time periods for the study duration was then calculated.

#### Step count

A total of 208 step counts made up the data set. IceTag (IceRobotics Ltd, South Queensferry, Edinburgh, Scotland, UK) accelerometers were placed on the left rear leg of each calf for the duration of the study. Accelerometer ID was paired with calf ID prior to placement onto the calf. Accelerometers were removed at the conclusion of the study and data was downloaded from the accelerometers for analysis.

#### Prostaglandin E_2_ metabolite

A total of 78 samples were analyzed for PGEM. Prostaglandin E_2_ metabolites were analyzed using a commercially available ELISA kit (cat. no. 514531, Cayman Chemical, Ann Arbor, MI) following manufacturer specifications with minor modifications. Sample input was adjusted to 375 µL with 1.5-mL ice-cold acetone added for sample purification. Samples were incubated at −20 °C for 30 min, then centrifuged at 3,000 × g for 5 min. Supernatant was transferred to clean 13 × 100 mm glass tubes and evaporated using a CentriVap Concentrator (cat. no. 7810014, Labconco, Kansas City, MO) overnight (approx. 18 h). Samples were reconstituted with 375 µL of appropriate kit buffer. A 300-µL aliquot of the reconstituted sample was derivatized with proportionally adjusted kit components. Manufacturer protocol was then followed. Samples were diluted 1:2 and ran in duplicate. Absorbance was measured at 405 nm after 60 min of development (SpectraMax i3, Molecular Devices, San Jose, CA). Sample results were excluded if the raw read exceeded the raw read of the highest standard (Standard 1; 50 pg/mL) or was below the lowest acceptable standard. The lowest acceptable standard was defined for each individual plate and was identified by excluding standards that had a ratio of absorbance of that standard to the maximum binding of any well (%B/B_0_) of ≥ 80% or ≤ 20%. Any individual sample outside the standard curve, with a %B/B_0_ outside the 20% to 80% range, or a coefficient of variation (**CV**) > 15% were re-analyzed.

#### Serum amyloid A

A total of 312 serum amyloid A samples made up the data set. Serum Amyloid A concentrations were determined in serum samples using an ELISA assay (Phase Range Multispecies SAA ELISA kit; Tridelta Development Ltd., Cat no. TP802). Manufacturer specifications were followed and samples were diluted as necessary. Absorbance was measured at 450 nm on a SpectraMax i3 plate reader (Molecular Devices). Raw data was analyzed using MyAssays Desktop software (version 7.0.211.1238) for concentration determination. Standard curves were plotted as a four-parameter logistic curve. Samples with a CV > 15% were re-analyzed.

#### Rectal temperature

A total of 260 rectal temperature samples made up the data set. Rectal temperatures were taken by placing a digital thermometer (180 Innovations, Lakewood, CO) against the wall of the rectum until a temperature reading was produced on the screen of the thermometer.

#### Receiver operating characteristic curve determination

All statistics were performed using statistical software (JMP Pro 14.0, SAS Institute, Inc., Cary, NC). Receiver operating characteristic curves were created for each time point, with AUC values comparing > 10% lung lesions to < 10%, with > 10% as the positive control. The biomarker was plotted as the x-coordinate and the status (>10% or < 10%) was plotted as the y-coordinate. Bootstrapping via fractional weights was used to generate confidence intervals for each AUC value. AUC values ≥ 0.7 were considered to yield good diagnostic accuracy (Yang and Berdine, 2017). Specific cutoff values were selected based upon optimized specificity and sensitivity values. Positive and negative predictive values with confidence intervals were calculated for each AUC value (MedCalc Software Ltd., Ostend, Belgium).

## Results

Sixteen of the 18 calves that were inoculated with *M. haemolytica* had lung lesion scores > 10%, with all 18 calves having extensive lung consolidation in their right apical lung lobe. Three of the eight control calves also had lung lesion scores > 10%, with four of the eight having extensive lung consolidation in their right apical lung lobe. Five calves received tildipirosin as an intervention and all had lung lesion scores > 10%. Seven of the eight calves who received flunixin had lung lesion scores > 10%. The biomarker parameter AUC values are outlined in Table 2, with rankings in Table 3 and cutoff values in Table 4.

**Table 2.** AUC values for lung scores < 10% vs. lung scores < 10% by collection time point for each biomarker

Biomarker^1^	Hour											
	0	4	8	24	32	48	72	96	120	144	168	192
Cortisol	0.64	0.59	0.63	0.60	0.66	0.63	0.64	0.77	0.69	0.68	0.81	0.67
IRT	0.74	0.64	0.69	0.59	0.71	0.64	0.58	0.69	0.66	0.69	0.67	0.75
MNT avg			0.60	0.69			0.67					0.64
MNT percent change			0.74	0.60			0.58					0.66
Substance P	0.71	0.69	0.63	0.68	0.65	0.64	0.61	0.61	0.62	0.59	0.64	0.59
Rf stance time	0.61	0.62	0.59	0.62	0.62	0.62	0.61	0.60	0.67	0.60	0.61	0.66
Rf stride length	0.81	0.64	0.67	0.79	0.62	0.65	0.72	0.70	0.62	0.63	0.60	0.61
Rf force	0.65	0.63	0.65	0.60	0.58	0.64	0.64	0.60	0.61	0.61	0.58	0.76
Rf impulse	0.59	0.62	0.61	0.65	0.61	0.60	0.59	0.59	0.64	0.62	0.59	0.73
Rf pressure	0.62	0.64	0.62	0.65	0.62	0.56	0.61	0.68	0.61	0.72	0.64	0.74
Gait distance	0.60	0.65	0.66	0.62	0.69	0.61	0.64	0.62	0.78	0.73	0.59	0.68
Gait velocity	0.58	0.61	0.62	0.68	0.77	0.70	0.66	0.67	0.62	0.60	0.59	0.61
VAS	0.78	0.68	0.70	0.68	0.67	0.84	0.68	0.68	0.73	0.75	0.75	0.73
CIS	0.65	0.58	0.60	0.59	0.60	0.77	0.62	0.72	0.64	0.68	0.64	0.67
Computerized lung score	0.59	0.58	0.58	0.68	0.61	0.58	0.56	0.71	0.64	0.57	0.64	0.56
Average activity	0.71			0.74		0.90	0.86	0.85	0.89	0.83	0.80	
Average rumination	0.63			0.58		0.81	0.68	0.68	0.73	0.68	0.69	
Step count	0.74			0.64		0.84	0.80	0.83	0.80	0.84	0.74	
PGEM	0.74						0.78					0.70
Serum Amyloid A	0.62	0.59	0.53	0.62	0.59	0.62	0.63	0.61	0.61	0.66	0.63	0.71
Rectal Temperature	0.72		0.69	0.78	0.63	0.90	0.82	0.66	0.61	0.57	0.66	

IRT, infrared thermography; MNT, mechanical nociceptive threshold; Rf, right front; VAS, visual analog scale; CIS, clinical illness score; PGEM, prostaglandin E_2_ metabolites.

**Table 3.** AUC value rankings > 0.7 for each biomarker by collection time point (h) with 0 h being the time of disease onset

Biomarker^1^	Ranking											
	1		2		3		4		5		6	
	AUC	Time point	AUC	Time point	AUC	Time point	AUC	Time point	AUC	Time point	AUC	Time point
Average activity	0.90	48	0.89	120	0.86	72	0.85	96	0.83	144	0.80	168
Rectal temperature	0.90	48	0.82	72	0.78	24	0.72	0				
Step count	0.84	48	0.84	144	0.83	96	0.80	72	0.80	120	0.74	168
VAS	0.84	48	0.78	0	0.75	144	0.75	168	0.73	120	0.73	192
Cortisol	0.81	168	0.77	96								
Rf stride length	0.81	0	0.79	24	0.72	72	0.70	96				
Gait distance	0.78	120	0.73	144								
PGEM	0.78	72	0.74	0	0.70	192						
CIS	0.77	48	0.72	96								
IRT	0.77	192	0.71	32								
Gait velocity	0.77	32	0.70	48								
Rf force	0.76	192										
MNT percent change	0.74	8										
Rf pressure	0.74	192	0.72	144								
Rf impulse	0.73	192										
Substance P	0.71	0										
Computerized lung score	0.71	96										
Serum amyloid A	0.71	192										

VAS, visual analog scale; Rf, right front; PGEM, prostaglandin E_2_ metabolites; CIS, clinical illness score; IRT, infrared thermography; Rf, right front; MNT, mechanical nociceptive threshold.

**Table 4.** Cutoff values for lung scores > 10% vs. lung scores < 10% by collection time point for each biomarker

Biomarker^1^	Units	Hour											
		0	4	8	24	32	48	72	96	120	144	168	192
Cortisol	ng/mL	9.72	14.54	2.84	11.75	1.8	6.12	5.02	1.17	2.64	2.69	3.82	1.03
IRT	°C	31.06	34.56	34.5	31.11	33.89	30.61	27.89	30.22	32.22	28.11	27.89	29.17
MNT avg	kg F			1.84	1.30			1.97					1.09
MNT percent change	%			0.23	-0.34			0.40					0.21
Substance P	pg/mL	388.52	409.71	473.29	374.61	129.31	360.21	344.13	185.86	113.19	368.81	146.49	484.05
Rf stance time	s	0.71	0.94	0.62	0.47	0.80	0.68	0.85	0.86	0.81	1.14	0.67	0.87
Rf stride length	cm	122.4	127.5	112.2	110.5	120.7	125.8	113.9	125.8	115.6	113.9	110.5	112.2
Rf force	kg	99.35	82.5	105.27	81.01	69.36	95.62	80.73	82.73	94.44	94.18	99.41	89.89
Rf impulse	kg∗s	60.24	71.21	50.74	32.58	46.8	35.61	62.69	50.85	35.69	50.17	36.62	45.83
Rf pressure	kg/cm^2^	4.7	7.3	5.2	5	6.2	5.8	4.8	4.8	4.6	7.8	5	4.8
Gait distance	cm	166.6	168.3	137.7	175.1	161.5	190.4	170.0	190.4	163.2	122.4	171.7	161.5
Gait velocity	cm/s	86.8	138.8	122.9	88.5	95.4	98.3	92.2	71.9	84.1	67.5	85.0	82.4
VAS	1–10 cm	8.5	6.5	9.0	6.5	6.0	6.0	5.5	8.5	9.5	7.0	11.0	8.5
CIS	0 = normal, ≥1 abnormal	1	1	1	1	1	1	1	1	1	1	1	1
Computerized lung score	0 = normal, ≥1 abnormal	1	0	0	1	0	0	0	1	1	0	1	0
Average activity	60 min average	27.67			23.00		23.33	23.58	21.58	22.75	25.75	19.55	
Average rumination	60 min average	37.33			13.17		23.92	44.50	37.33	42.67	33.08	26.64	
Step count	Count	1606			1549		1075	1271	1136	1352	1383	878	
PGEM	pg/mL	31.26						28.40					17.54
Serum amyloid A	μg/mL	423.73	419.50	484.64	321.17	251.03	305.07	237.07	132.47	73.07	54.68	77.28	55.36
Rectal temperature	°C	39.17		39.78	39.67	39.67	38.94	39.33	39.05	38.17	38.94	38.78	

IRT, infrared thermography; MNT, mechanical nociceptive threshold; Rf, right front; VAS, visual analog scale; CIS, clinical illness score; PGEM, prostaglandin E_2_ metabolites.

### Average activity and rumination

**Figure 1.**
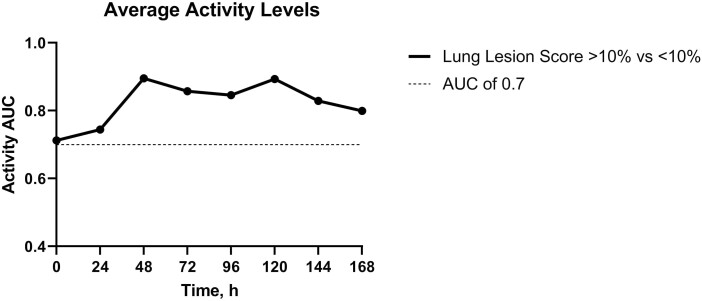
Area under the curve (AUC) values for average activity levels.

BRD study results comparing calves with significant lung lesions to those without yielded good diagnostic accuracy (AUC > 0.7; 95% CI: 0.70 to 0.90) for average activity at 0 h (cutoff: 27.67 min, PPV: 88.48%, NPV: 82.41%), 24 h (cutoff: 23 min, PPV: 100%, NPV: 38.98%), 48 h (cutoff: 23.33 min, PPV: 89.96%, NPV: 83.39%), 72 h (cutoff: 23.58 min, PPV: 90.44%, NPV: 100%), 96 h (cutoff: 21.58 min, PPV: 100%, NPV: 46.76%), 120 h (cutoff: 22.75 min, PPV: 94.10%, NPV: 66.75%), 144 h (cutoff: 25.75 min, PPV: 86.32%, NPV: 100%), and 168 h (cutoff: 19.55 min, PPV: 100%, NPV: 43.85%; Figure 1). BRD study results comparing calves with significant lung lesions to those without yielded good diagnostic accuracy (AUC > 0.7; 95% CI: 0.72 to 0.82) for average rumination at 48 h (cutoff: 23.92 min, PPV: 100%, NPV: 43.85%) and 120 h (cutoff: 42.67 min, PPV: 88.19%, NPV: 55.65%).

### Step count

**Figure 2.**
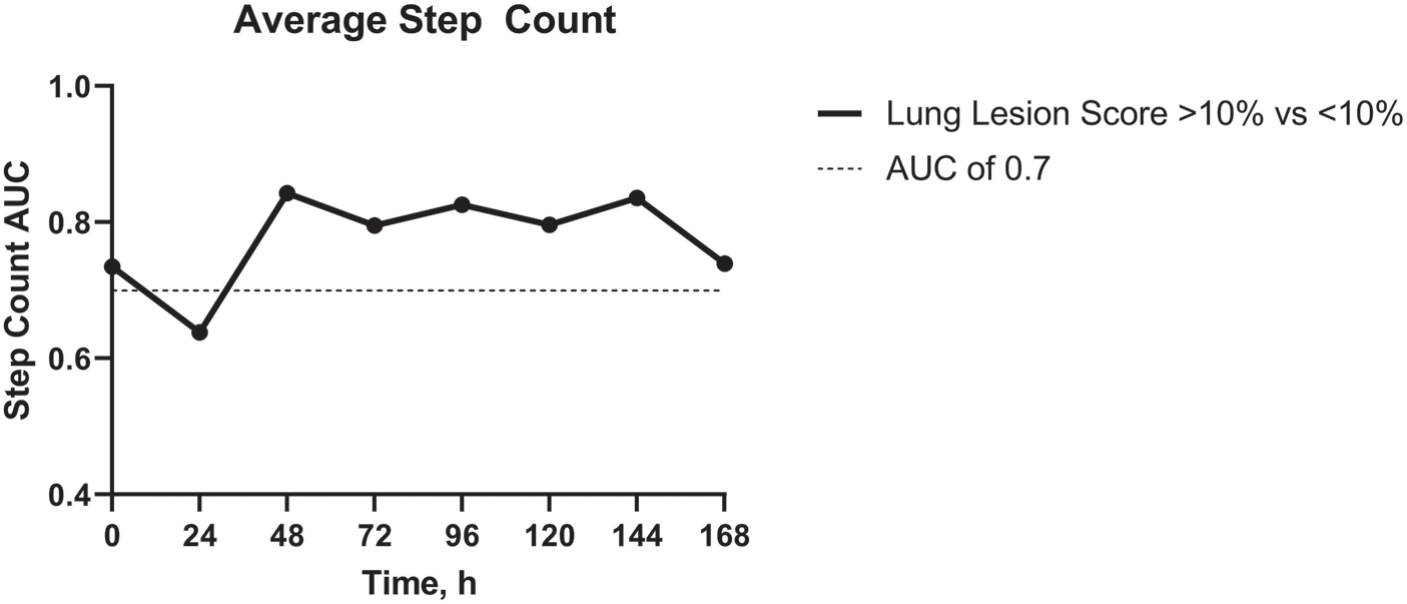
Area under the curve (AUC) values for average step count.

BRD study results comparing calves with significant lung lesions to those without yielded good diagnostic accuracy (AUC > 0.7; 95% CI: 0.73 to 0.85) for average step count at 0 h (cutoff: 1606 steps, PPV: 88.75%, NPV: 61.32%), 48 h (cutoff: 1075 steps, PPV: 90.44%, NPV: 100%), 72 h (cutoff: 1271 steps, PPV: 90.44%, NPV: 100%), 96 h (cutoff: 1136 steps, PPV: 75.81%, NPV: 35.17%), 120 h (cutoff: 1352 steps, PPV: 90.44%, NPV: 100%), 144 h (cutoff: 1383 steps, PPV: 90.44%, NPV: 100%), and 168 h (cutoff: 878 steps, PPV: 85.63%, NPV: 79.19%; Figure 2).

### Rectal temperature

BRD pain study results comparing calves with significant lung lesions to those without yielded good diagnostic accuracy (AUC > 0.7; 95% CI: 0.71 to 0.90) for rectal temperature at 0 h (cutoff: 39.17 °C, PPV: 86.49%, NPV: 53.94%), 24 h (cutoff: 39.67 °C, PPV: 92.83%, NPV: 50.10%), 48 h (cutoff: 38.94 °C, PPV: 93.73%, NPV: 60.09%) and 72 h (cutoff: 39.33 °C, PPV: 100%, NPV: 46.76%).

### Visual analog scale

**Figure 3.**
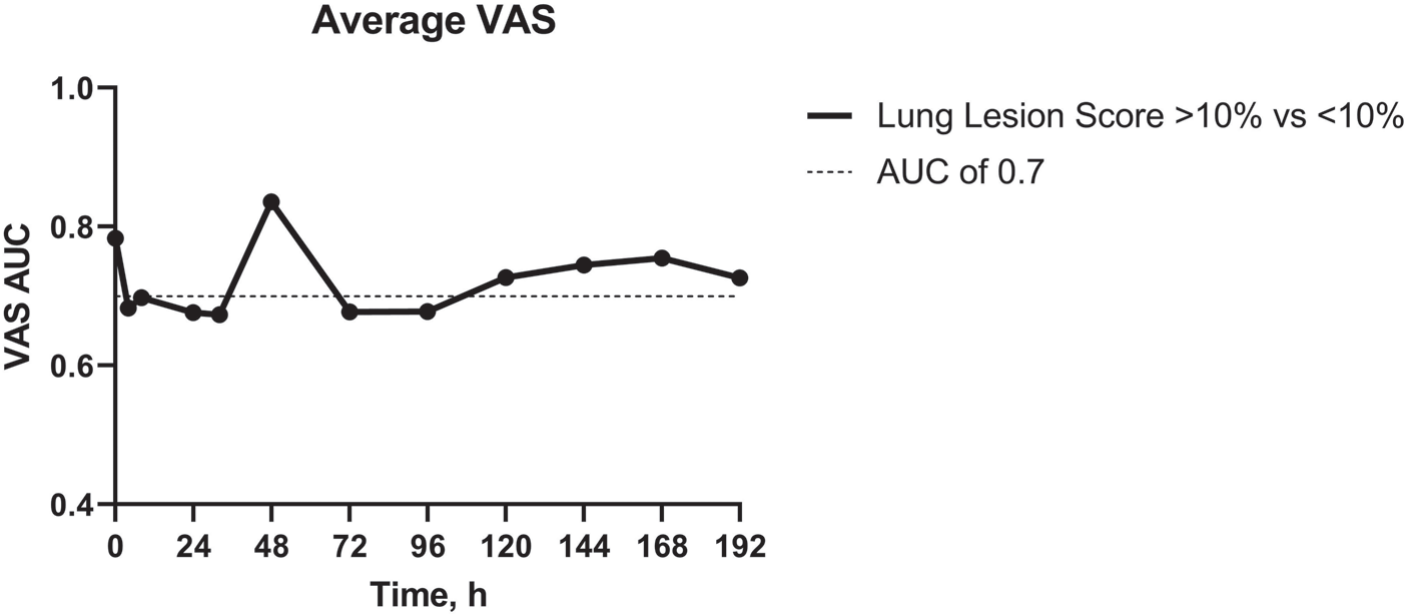
Area under the curve (AUC) values for visual analog scale (VAS) scores.

BRD study results comparing calves with significant lung lesions to those without yielded good diagnostic accuracy (AUC > 0.7; 95% CI: 0.72 to 0.84) for VAS score at 0 h (cutoff: 8.5, PPV: 92.28%, NPV: 53.94%), 48 h (cutoff: 6, PPV: 90.44%, NPV: 100%), 120 (cutoff: 9.5, PPV: 100%, NPV: 41.27%), 144 h (cutoff: 7, PPV: 88.85%, NPV: 62.59%), 168 h (cutoff: 1, PPV: 91.64%, NPV: 42.95%), and 192 h (cutoff: 8.5, PPV: 84.16%, NPV: 57.24%; Figure 3).

### Serum cortisol

BRD study results comparing calves with significant lung lesions to those without yielded good diagnostic accuracy (AUC > 0.7; 95% CI: 0.76 to 0.81) for cortisol at 96 h (cutoff: 1.17 ng/mL, PPV: 92.06%, NPV: 67.44%) and 168 h (cutoff: 3.82 ng/mL, PPV: 100%, NPV: 66.36%).

### Kinematic gait analysis

BRD study results comparing calves with significant lung lesions to those without yielded good diagnostic accuracy (AUC > 0.7; 95% CI: 0.70 to 0.82) for right front stride length at 0 h (cutoff: 122.4 cm, PPV: 92.26%, NPV: 71.55%), 24 h (cutoff: 110.5 cm, PPV: 100%, NPV: 45.42%), 72 h (cutoff: 113.9 cm, PPV: 88.14%, NPV: 55.21%), and 96 h (cutoff: 125.8 cm, PPV: 81.84%, NPV: 100%).

BRD study results comparing calves with significant lung lesions to those without yielded good diagnostic accuracy (AUC > 0.7; 95% CI: 0.75 to 0.76) for right front force at 192 h (cutoff: 89.89 kg, PPV: 88.63%, NPV: 59.95%).

BRD study results comparing calves with significant lung lesions to those without yielded good diagnostic accuracy (AUC > 0.7; 95% CI: 0.73 to 0.74) for right front impulse at 192 h (cutoff: 45.83 kg∗s, PPV: 88.63%, NPV: 59.95%).

BRD study results comparing calves with significant lung lesions to those without yielded good diagnostic accuracy (AUC > 0.7; 95% CI: 0.73 to 0.74) for right front pressure at 144 h (cutoff: 7.8 kg/cm^2^, PPV: 84.39%, NPV: 100%) and 192 h (cutoff: 4.8 kg/cm^2^, PPV: 100%, NPV: 41.13%).

BRD study results comparing calves with significant lung lesions to those without yielded good diagnostic accuracy (AUC > 0.7; 95% CI: 0.73 to 0.79) for gait distance at 120 h (cutoff: 163.2 cm, PPV: 88.63%, NPV: 59.95%) and 144 h (cutoff: 122.4 cm, PPV: 83.67%, NPV: 77.85%).

BRD study results comparing calves with significant lung lesions to those without yielded good diagnostic accuracy (AUC > 0.7; 95% CI: 0.73 to 0.79) for gait velocity at 32 h (cutoff: 95.4 cm/s, PPV: 93.83%, NPV: 50.10%) and 48 h (cutoff: 98.3 cm/s, PPV: 84.16%, NPV: 57.24%).

### Prostaglandin E2 metabolite

**Figure 4.**
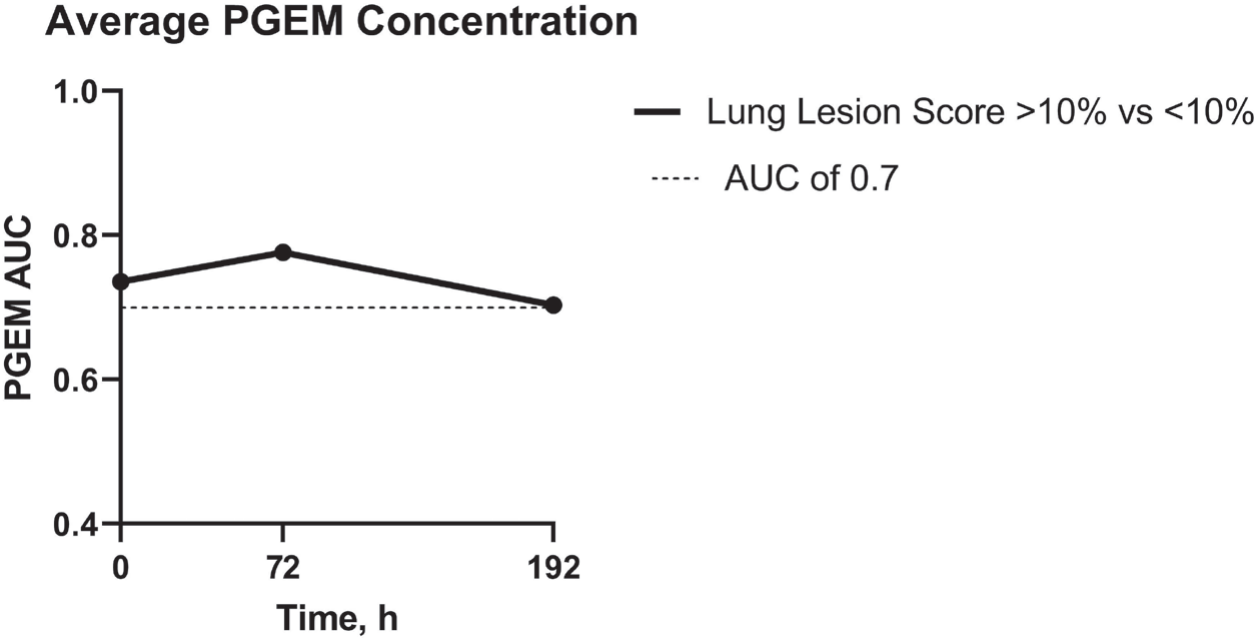
Area under the curve (AUC) values for prostaglandin E_2_ metabolite (PGEM) concentration.

BRD study results comparing calves with significant lung lesions to those without yielded good diagnostic accuracy (AUC > 0.7; 95% CI: 0.70 to 0.78) for PGEM concentration at 0 h (cutoff: 31.26 pg/mL, PPV: 87.23%, NPV: 60.96%), 72 h (cutoff: 28.4 pg/mL, PPV: 100%, NPV: 41.27%) and 192 h (cutoff: 17.54 pg/mL, PPV: 88.19%, NPV: 55.65%; Figure 4).

### Clinical illness score

BRD pain study results comparing calves with significant lung lesions to those without yielded good diagnostic accuracy (AUC > 0.7; 95% CI: 0.72 to 0.78) for CIS at 48 h (cutoff: 1, PPV: 92.83%, NPV: 50.10%) and 96 h (cutoff: 1, PPV: 91.64%, NPV: 42.95%).

### Infrared thermography

BRD study results comparing calves with significant lung lesions to those without yielded good diagnostic accuracy (AUC > 0.7; 95% CI: 0.70 to 0.75) for IRT at 0 h (cutoff: 31.06 °C, PPV: 87.23%, NPV: 60.96%), 32 h (cutoff: 33.89 °C, PPV: 100%, NPV: 38.98%), and 192 h (cutoff: 29.17 °C, PPV: 37.45%, NPV: 80.06%).

### Mechanical nociception threshold

BRD study results comparing calves with significant lung lesions to those without yielded good diagnostic accuracy (AUC > 0.7; 95% CI: 0.70 to 0.75) for MNT percent change from baseline at 8 h (cutoff: 0.23 kg F, PPV: 88.19%, NPV: 55.65%).

### Substance P

BRD study results comparing calves with significant lung lesions to those without yielded good diagnostic accuracy (AUC > 0.7; 95% CI: 0.70 to 0.71) for substance P at 0 h (cutoff: 388.52 pg/mL, PPV: 90.38%, NPV: 42.26%).

### Computerized lung score

BRD pain study results comparing calves with significant lung lesions to those without yielded good diagnostic accuracy (AUC > 0.7; 95% CI: 0.70 to 0.71) for CLS at 96 h (cutoff: 1, PPV: 84.16%, NPV: 57.24%).

### Serum amyloid A

BRD pain study results comparing calves with significant lung lesions to those without yielded good diagnostic accuracy (AUC > 0.7; 95% CI: 0.70 to 0.71) for SAA concentrations at 192 h (cutoff: 55.36 μg/mL, PPV: 89.44%, NPV: 71.51%).

## Discussion

The BRD induction model used in the present study was expected to produce pleuritis, and gross necropsy findings revealed that pleuritis was present in inoculated calves with significant lung consolidation. Cattle with advanced lung lesions at harvest have been shown to have reduced average daily gain; hot carcass weight; kidney, pelvic, and heart fat; 12th rib fat; calculated yield grades; marbling scores and percentage choice carcasses (Schneider et al., 2009; Tennant et al., 2014; Blakebrough-Hall et al., 2020), all resulting in economic losses. This creates a need for the development of robust biomarkers to objectively predict BRD and the presence of lung lesions.

Biomarkers previously used as diagnostic tools for BRD diagnosis include cortisol concentrations (Theurer et al., 2013; Foote et al., 2017), infrared thermography (Baruch et al., 2019), computerized lung score (Zeineldin et al., 2016; Baruch et al., 2019; Nickell et al., 2020), acute-phase protein levels (Abdallah et al., 2016), clinical illness score (Amrine et al., 2013), and activity and behavior measurements (Hanzlicek et al., 2010; White et al., 2012; Theurer et al., 2013; Pillen et al., 2016; Tomczak et al., 2019).

Accelerometers have been used to monitor animal behavior without the presence of human evaluators which can reduce subjectivity (White et al., 2008). Our results showed the highest AUC values to be associated with average activity levels and step count indicating that they may yield the best diagnostic accuracy. Diagnostic accuracy remained strong throughout the duration of the study, indicating that activity levels and step count may be viable options for quantifying differences early in the BRD disease process as well as during the disease progression.

Rectal temperatures yielded good diagnostic accuracy, particularly between 24 and 72 h after disease onset. In previous BRD challenge studies, rectal temperature differed between days when animals were diseased when compared with healthy (Baruch et al., 2019) and calves exhibited fever as part of the sickness response on days 3 to 7 (Toaff-Rosenstein et al., 2016).

Visual analog scale assessment is a method of evaluating pain intensity based on behavioral parameters. Our results revealed good diagnostic accuracy for VAS values out to 9 d following inoculation which may be due to the nature of BRD causing more chronic changes that VAS scoring captured 7 to 9 d postinoculation. Cutoff values did not decrease throughout the study, supporting the idea that calves were painful for the study duration.

Cortisol results from the present study revealed higher AUC values later in the study indicating that there may be meaningful differences in cortisol levels more chronically and less acutely when identifying calves with lung lesions. These results are similar to our previous findings for AUC values for lameness which is also a more chronic condition (Kleinhenz et al., 2019b). Cutoff values were lower than cutoff values from previous findings, indicating that BRD may not cause as acute or magnified of a cortisol response as some procedures such as castration and dehorning.

Kinematic gait analysis in cattle has not been well-characterized in the past but is becoming more prevalently used as a diagnostic tool (Pairis-Garcia et al., 2015; Kleinhenz et al., 2018, 2019a). A commercially available floor mat–based gait system can be used to assess variables such as gait distance and weight distribution (Coetzee et al., 2017). Our results yielded good diagnostic accuracy for right front stride length. In the present study, the right apical lung lobes were inoculated indicating that gait measurements more specific to the area of trauma may yield better results.

Bacterial infections such an pneumonia cause the activation of monocytes and macrophages and release of inflammatory mediators such as prostaglandin E_2_ (Idoate et al., 2015). Our results revealed good diagnostic accuracy for PGEM concentrations with cutoff values decreasing over the duration of the study. Concentrations of PGEM were only quantified at three time points throughout the study which all yielded good diagnostic accuracy, at 0, 72, and 192 h indicating that PGEM concentrations maintained good diagnostic accuracy throughout the study.

Clinical illness scores yielded good diagnostic accuracy 48 and 96 h after BRD onset but did not continue to yield good diagnostic accuracy throughout the study duration. Amrine et al. (2013) found that interobserver agreement for assigning CIS was low and CIS accuracy relative to pulmonary consolidation varied based upon the severity of consolidation.

The AUC values from this analysis did not indicate a clear pattern for when IRT may yield good diagnostic accuracy. An acute epinephrine release would be more likely to occur shortly after an acutely painful procedure such as dehorning relative to respiratory disease event. Environmental factors along with distance from the animal can be very influential upon IRT readings (Church et al., 2014). Sampling time points being at different points throughout the day resulted in varying ambient temperatures that likely influenced results.

Determining mechanical nociception threshold via a pressure algometer can establish the minimal amount of pressure that produces a response. The AUC values indicated poorer diagnostic accuracy relative to previous findings when MNT was used in a dehorning study (Kleinhenz et al., 2017). Our results also revealed higher cutoff values relative to dehorning cutoff values. This may be due to calves being more sensitive to force around their horn buds compared to calves being relatively less sensitive to force in their thoracic region.

Our results showed poor diagnostic accuracy for substance P when identifying calves with lung lesions. Previous cattle pain studies did not find a difference in substance P levels between calves likely experiencing pain from procedures such as dehorning and castration compared with sham calves who likely were not in pain (Kleinhenz et al., 2017, 2018). Higher cutoff values were observed in our results relative to previous findings indicating that BRD may result in higher substance P levels relative to castration and dehorning studies (Kleinhenz et al., 2017, 2018).

Computerized lung scores only seemed to yield good diagnostic accuracy at 96 h after BRD onset. Baruch et al. (2019) when examining the association between computerized lung score relative to lung consolidation found a trend toward significance with a difference only between normal and moderate acute scores. This analysis did not differentiate between mild and moderate acute CLS. This analysis used a cutoff of < 10% consolidation while (Baruch et al., 2019) found a mean of 13.7% lung consolidation to be associated with a normal CLS score.

Our results yielded poor diagnostic accuracy for serum amyloid A concentrations which was likely due to all calves having elevated SAA levels regardless of lung lesions. Higher levels of SAA were observed in the present study compared to levels in previously recorded naturally occurring BRD in calves (Joshi et al., 2018). In the present study, the cutoff values were most elevated at 8 h and steadily decreased for the remainder of the study, indicating that a SAA response was observed.

The impact on animal well-being from BRD is considerable (Hanzlicek et al., 2010). Pleuritic chest pain as a result of bacterial pneumonia is commonly reported in human medicine (Boyd et al., 2006); however, published literature regarding pain associated with lung lesions as a result of bacterial pneumonia in cattle is lacking. The biomarkers quantified in the present study varied in their specificity to BRD, objectivity, and association with pain. Through previous ROC analysis we identified cortisol, hair cortisol and infrared thermography imaging as having acceptable (AUC > 0.7) diagnostic sensitivity and specificity for detecting pain in cattle (Martin et al., 2020b). In the present study, average activity levels yielded good diagnostic accuracy for predicting lung lesions and were objective, but not specific to pain. Visual analog scale scores yielded good diagnostic accuracy and were somewhat specific to pain but were not specific to BRD and were subjective. Rectal temperatures yielded good diagnostic accuracy and were objective but were not specific to BRD or pain. Identifying biomarkers that are (1) predictive of lung lesions and (2) specific to pain will require further investigation into diagnostic tools and biomarkers to quantify pain from BRD.

## Conclusions

In the first 72 h after onset of BRD, right front stride length, gait velocity, VAS, CIS, average activity level, step count, and rectal temperature yielded the best diagnostic accuracy (AUC > 0.75) for predicting calves with significant lung lesions (>10% consolidation) at necropsy compared with those with < 10% lung lesions. After 72 h postinduction, PGEM, gait distance, cortisol, IRT, right front force, average activity level, step count, and serum amyloid A yielded the best diagnostic accuracy (AUC > 0.75) for predicting the severity of lung lesions. Biomarkers and clinical signs with the best diagnostic accuracy early in the disease process would likely be the most valuable in field conditions. These results indicate that ROC analysis can be a useful indicator of the predictive value of biomarkers and clinical signs associated with pain and inflammation in cattle with induced bacterial pneumonia. AUC values for VAS score, average activity levels, step count, and rectal temperature seemed to yield very good diagnostic accuracy (AUC > 0.75) at multiple time points, while average and percent change MNT values, substance P concentrations, and CLS did not (all AUC values < 0.75). These results can be used to guide refinement of the optimal time points and biomarkers for the diagnosis of significant lung lesions after BRD.

## Abbreviations

AUC: area under the curve
BRD: bovine respiratory disease
CIS: clinical illness score
CLS: computerized lung score
IRT: infrared thermography
MNT: mechanical nociceptive threshold
PGEM: prostaglandin E2 metabolite
ROC: receiver operating characteristic
SAA: serum amyloid A
VAS: visual analog scale
